# Investigating health related quality of life and clinical measures in autoimmune encephalitis: a systematic review

**DOI:** 10.1186/s13023-025-03837-7

**Published:** 2025-06-13

**Authors:** Leonard Lee, Jovi Leung, Brendan Min-Wei Chan, Joshua Byrnes, Hansoo Kim

**Affiliations:** 1 Centre for Applied Health Economics, School of Medicine and Dentistry, Griffith University, 1 Parklands Drive, Southport, QLD 4215 Australia; 2 Faculty of Medicine, University of Queensland, Brisbane, Australia; 3 Faculty of Medicine and Health, University of Sydney, Sydney, Australia

**Keywords:** Autoimmune encephalitis (AE), Quality of life (QoL), Disease burden, Systemic review

## Abstract

This systematic review sought to understand the types of quality of life or clinical outcome measures currently being utilised for autoimmune encephalitis (AE) patients to determine whether the current measured outcomes accurately represented the full disease burden of AE. This included how the measures were being used (e.g., time point utilised, as an endpoint or as a categorisation method), and what the recorded measures were. The review included all studies that fulfilled the population criteria, which included probable AE. A search conducted on the 24th of June 2024 on PubMed and Embase returned 302 studies that fulfilled the inclusion criteria. Of the 302, the most commonly used measures were the Modified Rankin Scale (mRS) being utilised in 90.07% of studies, followed by Clinical Assessment Scale in Autoimmune Encephalitis (CASE) at 15.56%, Montreal Cognitive Assessment (MoCA) at 7.95%, and finally Mini Mental State Examination (MMSE) at 4.97%. The most common phenotype in the literature was NMDA AE, with 208 studies (68.87%) investigating this cohort. There was significant heterogeneity in the application of measures, such as definitional differences (e.g., a good outcome defined as an mRS of < 1 for some papers, but < 2 for others), utilising measures at differing time points within the disease course of an individual, and the inconsistent reporting of key information, such as comorbidities that may impact the measures being used. This review found the current clinical measures do not holistically encompass the disease burden of AE, *with current clinical measures experiencing deficiencies in capturing the effect of AE on sleep and psychosocial function*. It is recommended that future studies utilise symptom onset as a standardised time metric, and either a combination of currently available measures, or the creation or validation of new methods to capture disease burden in AE patients.

**Supplementary Information:**

The online version contains supplementary material available at 10.1186/s13023-025-03837-7.

## Background

Acute encephalitis is a debilitating neurological condition that significantly burdens patients and their families [1–5]. Historically, the frequency of diagnosing infectious origins have resulted in treatment guidelines that neglect autoimmune aetiologies [5, 6]. However, with the increased recognition of autoimmune encephalitis (AE), re-evaluations to criteria are emerging such as a 2016 position paper by an expert panel made of clinicians with extensive knowledge of AE [3]. Due to the heterogeneous nature of AE, various categorisations of the different aetiologies exist. Nevertheless, a serology grouping utilising the target of the AE antibody is popular, as this provides guidance on the treatment response, association with underlying malignancy, and long-term prognosis [1, 7]. Epidemiological data on AE is likely under-reported due to the heterogeneous presentations and uncertain mechanism of action, and very few epidemiological studies exist. For instance, recent incidence estimates of intracellular AE suggest a true rate likely much higher than the previous consensus of < 1% [7, 8]. Further, an incidence estimate of 13.7 per 100,000 was reported by a study from Olmsted County, Minnesota, while the Association of British Neurologists and British Infection Association National Guidelines on encephalitis estimated the incidence to be between 0.7 and 12.6 per 100,000 with different sub-categorisations of AE disproportionately impacting different population groups [9, 10].

The current diagnostic criteria for AE from the 2016 expert review by Graus et al. requires the presence of clinical presentations, imagining abnormalities, and the exclusion of alternative causes [3]. However, due to the novel and rare nature of AE, diagnosis can be complex, with a wide differential and uncertainty surrounding disease aetiology and disease course, complicating clinician responses. For example, clinical presentations of AE differ both between and within phenotypes and are dependent on the involved antigens and CNS regions affected [1, 2, 4]. Presentations can include movement disorders, cognitive impairment, psychiatric symptoms and seizures [3, 11–16]. In elderly patients, AE also typically presents with rapidly progressive dementia and significant memory decline [17]. Paediatric cases are more likely than adults to have seizures (commonly tonic-clonic and focal), atypical motor symptoms (such as hemiparesis or ataxia), and abnormal movements earlier in the disease course, with behavioural regression more common than the psychosis presentation in adults [18]. Generally for AE, symptoms can be acute or subacute (weeks to months) and can be progressive in nature with neurocognitive symptoms such as neuropsychiatric symptoms, brain stem syndromes, dysautonomia, seizures, encephalopathy, movement disorders, and cognitive dysfunction [1–3]. Additionally, symptoms can re-emerge later in life after initial acute presentations have disappeared [19–21].

Current therapeutic guidelines are predominantly formed from expert opinion and case series [1, 21]. Recommendations include early interventions and escalation to second-line immunotherapy to improve clinical outcomes. Gaps exist such as timeframes of optimal immunotherapy and how long clinicians should wait for the patient to respond to first-line immunotherapy [1, 22, 23]. First line treatment typically includes corticosteroids with intravenous immunoglobulin (IVIg) and plasmapheresis [1, 7, 22–24]. Utilisation of corticosteroids is problematic due to the difficulty with differentiating between infectious and autoimmune encephalitis in the acute stage, and the limited therapeutic effects on antibody titer [25, 26]. Further, while T cells (protective effect against infection) are reduced, the reduction on circulating B cells (which create antibodies), is sizably less, thus necessitating complementary immunotherapeutic against specifically targeting Ig and B cells [25]. As such, second line and maintenance treatments utilise other immunosuppressants such as cyclophosphamide, and monoclonal antibodies (e.g., rituximab) [1, 7, 22–24]. Finally, novel AE specific therapeutics are currently in development and under clinical trials, such as satralizumab, rozanolixizumab, bortezomib, inebilizumab, and natalizumab [28].

Autoimmune encephalitis is a devastating condition, compounded by its novelty and heterogenous disease course. Disease burden extends beyond the symptoms into many domains of life, such as anxiety and depression from the uncertainty of diagnosis and treatment to the detrimental impacts to relationships with family and friends. The range of impacts to quality of life (QoL) and activities of daily living (ADL) are broad, and an accurate picture can require more than one measure. Special consideration must be placed on accurate recognition of these detriments to QoL and ADL to allow for appropriate management and support. As such, this paper aims to assess the most common measures for QoL in the AE cohort, *whether these measures are sufficient to capture their holistic experience*, and *what alterations could improve assessment of the QoL in patients with AE.*

## Methods

A literature review of published journals on the QoL for patients living with AE was conducted given the limited nature of QoL research in AE cohorts (including suspected AE), with broadly applicable search terms given the varying disease states and novel nature of AE. On the 12th of July 2023, an initial search to gather common terms used within this study area was conducted through PubMed. Specifically, an initial pilot literature search with keyword searching for quality of life and clinical trials for AE was conducted, and all measures that were found in more than one study was included in the quality-of-life or clinical outcome measures search term. This was followed by cited reference searching. This was also performed to select the phenotypes included in the AE search string, however with a threshold of appearance in any study (rather than more than one). Then, the final search was performed on the 24th of June 2024 on PubMed and Embase, using medical subject headings (MeSH) and keywords for studies published between 2000 and 2024. Terms for quality of life (e.g., EQ-5D-5 L) and autoimmune encephalitis were used to create a complete search string, which appears in Tables 1 and 2. The desired outcome was any publications that presented any measures that related to QoL or clinical outcomes in AE.

We primarily sought to answer the following research questions:

1. What are the types of quality of life or clinical outcome measures being used for AE patients, and how are they being used?
2. How are they being used in this population, are there any considerations of generalisability for measures not validated in AE, and whether there are any differences in sensitivity/specificity, validity, reliability, responsiveness exists between subgroups?
3. What are the *current gaps in holistic quality of life assessments* for AE patients?

**Table 1:** Search terms used for literature search of the pubmed database (June 24th, 2024)

Thematic group	Search number	Search terms	Number of results
Quality of life or clinical outcome measures	1	(((“edss” OR (“expanded” AND “disability” AND “status” AND “scale”) OR “mrs” OR (“modified” AND “rankin” AND “score”)) OR ((“clinical” AND “assessment” AND “score”) OR “rbans” OR (“repeatable” AND “battery” AND “of” AND “neuropsychological” AND “status”) OR “moca” OR (“montreal” AND “overall” AND “cognitive” AND “assessment”) OR “wais” OR (“weschler” AND “adult” AND “intelligence” AND “scale”) OR “wms” OR (“weschler” AND “memory” AND “scale”))) OR (“hads” OR (“hospital” AND “anxiety” AND “depression” AND “scale”)) OR (“neuro-qol” OR “eq-5d-5l” OR “sf-36” OR “EuroQol 5 dimensions” OR (“36-item” AND “short” AND “form” and “survey”)) OR “quality of life”)	635,692
Autoimmune encephalitis	2	((“autoimmune encephalitis”) OR (“encephalitis” AND (“NMDA” OR “GABA” OR “LGI1” OR “AMPA” OR “DPPX” OR “CASPR2” OR “GlyR” OR “GAD65” OR “Ma2” OR “Hu” OR “PCA-1” OR “ANNA” OR “DNER” OR “VGCC”)))	5,907
Total	3	#1 AND #2	334

**Table 2:** Search terms used for literature search of the EMBASE database (June 24th, 2024)

Thematic group	Search number	Search terms	Number of results
Quality of life measures	1	(‘edss’:ab, ti OR (‘expanded’:ab, ti AND ‘disability’:ab, ti AND ‘status’:ab, ti AND ‘scale’:ab, ti) OR ‘mrs’:ab, ti OR (‘modified’:ab, ti AND ‘rankin’:ab, ti AND ‘score’:ab, ti) OR (‘clinical’:ab, ti AND ‘assessment’:ab, ti AND ‘score’:ab, ti) OR ‘rbans’:ab, ti OR (‘repeatable’:ab, ti AND ‘battery’:ab, ti AND ‘of’:ab, ti AND ‘neuropsychological’:ab, ti AND ‘status’:ab, ti) OR ‘moca’:ab, ti OR (‘montreal’:ab, ti AND ‘overall’:ab, ti AND ‘cognitive’:ab, ti AND ‘assessment’:ab, ti) OR ‘wais’:ab, ti OR (‘weschler’:ab, ti AND ‘adult’:ab, ti AND ‘intelligence’:ab, ti AND ‘scale’:ab, ti) OR ‘wms’:ab, ti OR (‘weschler’:ab, ti AND ‘memory’:ab, ti AND ‘scale’:ab, ti) OR ‘hads’:ab, ti OR (‘hospital’:ab, ti AND ‘anxiety’:ab, ti AND ‘depression’:ab, ti AND ‘scale’:ab, ti) OR ‘neuro-qol’:ab, ti OR ‘eq-5d-5l’:ab, ti OR ‘sf-36’:ab, ti OR ‘euroqol 5 dimensions’:ab, ti OR (‘36-item’:ab, ti AND ‘short’:ab, ti AND ‘form’:ab, ti AND ‘survey’:ab, ti) OR ‘quality of life’:ab, ti)	549,217
Autoimmune encephalitis	2	(‘autoimmune encephalitis’:ab, ti OR (‘encephalitis’:ab, ti AND (‘nmda’:ab, ti OR ‘gaba’:ab, ti OR ‘lgi1’:ab, ti OR ‘ampa’:ab, ti OR ‘dppx’:ab, ti OR ‘caspr2’:ab, ti OR ‘glyr’:ab, ti OR ‘gad65’:ab, ti OR ‘ma2’:ab, ti OR ‘hu’:ab, ti OR ‘pca-1’:ab, ti OR ‘anna’:ab, ti OR ‘dner’:ab, ti OR ‘vgcc’:ab, ti)))	6,650
Total	3	#1 AND #2	484

## Results

**Fig. 1:**
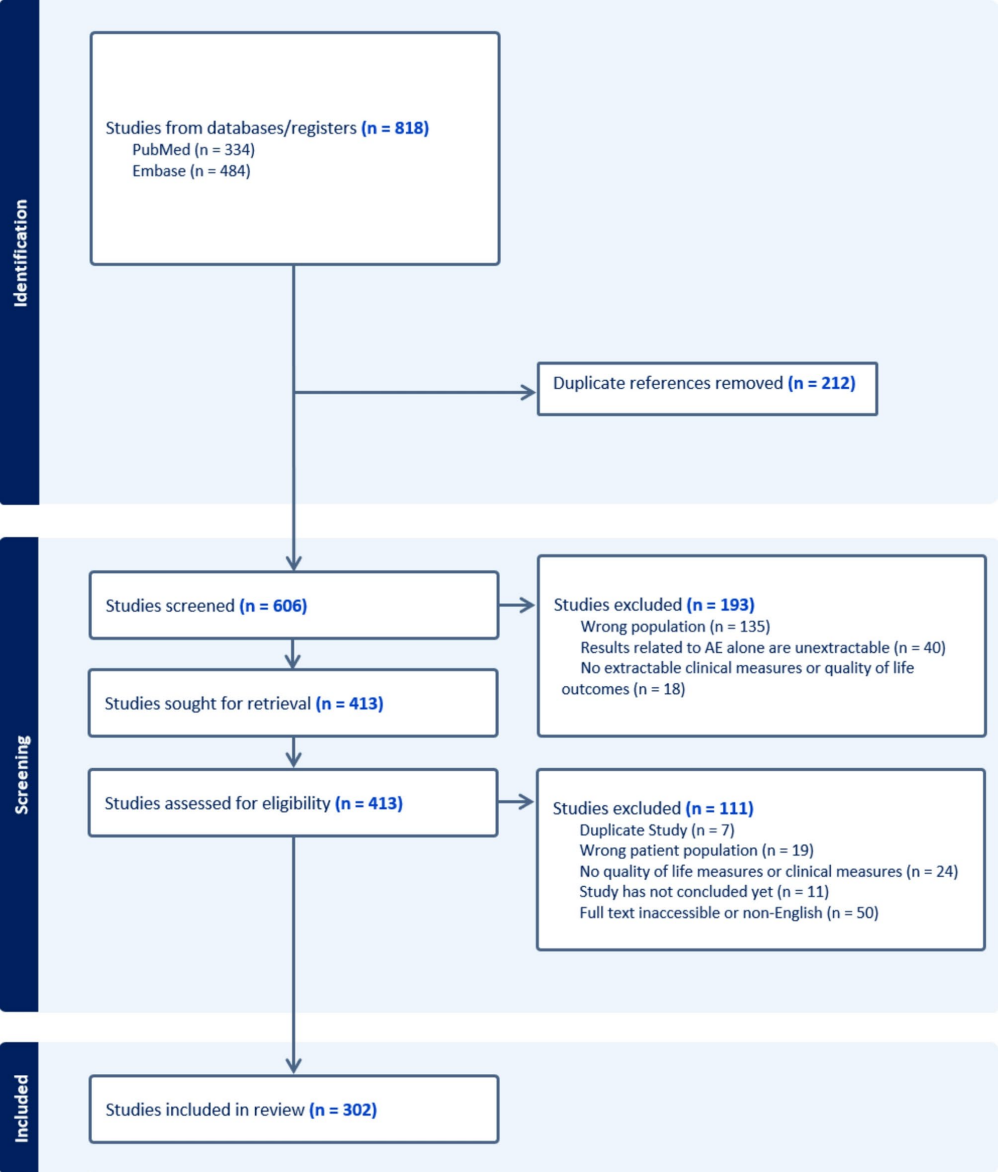
PRISMA diagram. Study selection for inclusion in the review

The search yielded 606 results after duplicates were removed, and during abstract screening, 135 were excluded for wrong population (i.e., not AE), 40 because outcomes only to related to AE was unextractable, and 18 did not have extractable clinical measures or quality of life outcomes. Of the remaining 413, during full text screening 111 studies were further excluded for the reasons presented below in Fig. 1.

**Fig. 2:**
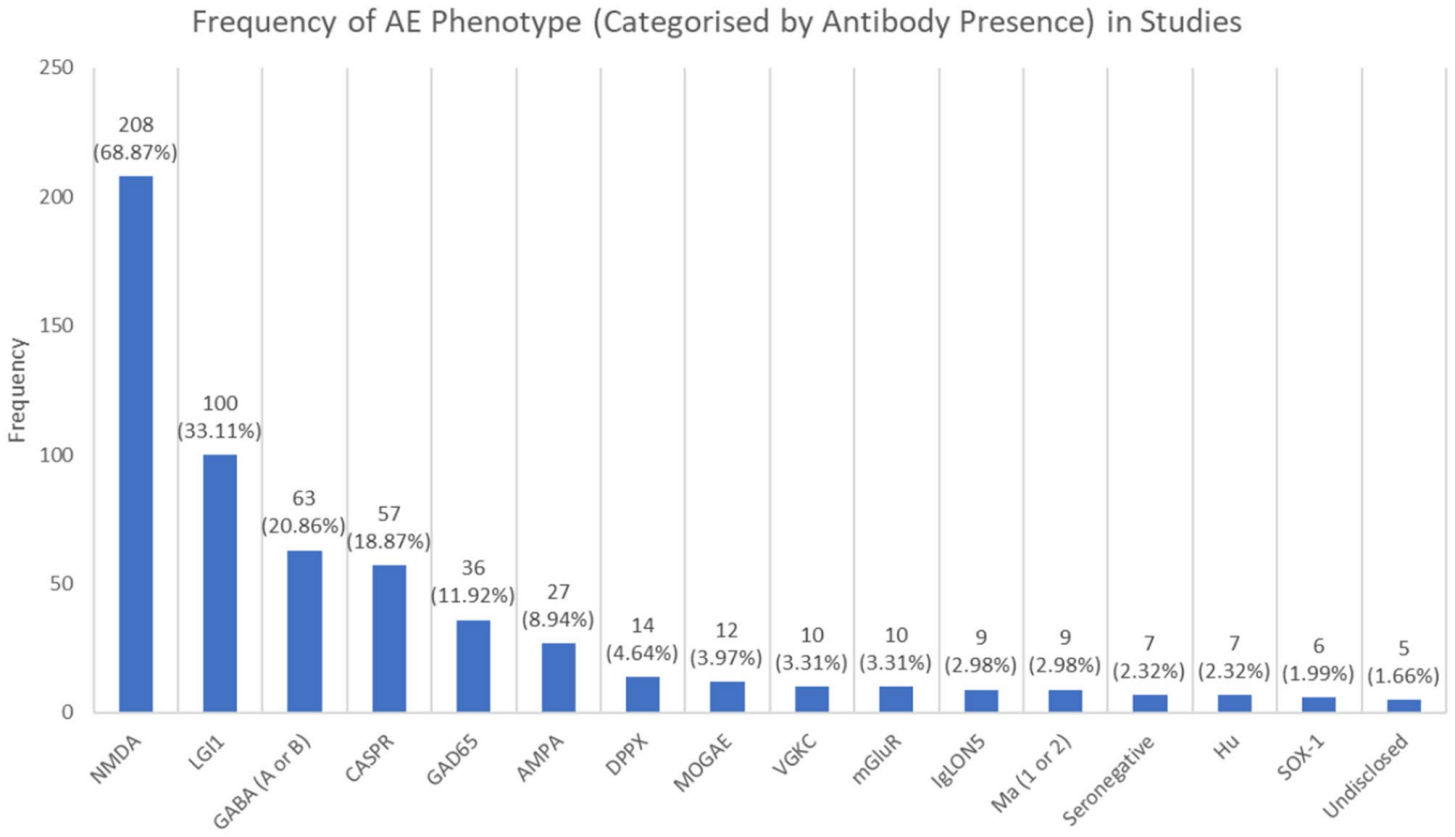
Frequency of AE phenotype (categorised by antibody presence) in studies

Figure 2 summarises the frequency of AE phenotype (categorised by antibody presence) in studies analysed. Phenotypes ≥ 5 are summarised, refer to supplementary material 1 for complete list.

The AE phenotype by frequency was predominantly NMDA AE (68.87%), then LGI1 AE (33.11%), GABA A or B (20.86%), CASPR (18.87%) and GAD65 (11.92%). No studies were randomised controlled trials, and of the single centred studies (*n* = 224, 74.17%), the mean number of participants (range) was 53.38 (1-343), while for multi-centred studies (*n* = 67, 22.91%) the mean number of participants was 139.67 (3-1550). Site type was undisclosed in seven studies. Complete demographics for each study can be found in supplementary material 1.

**Fig. 3:**
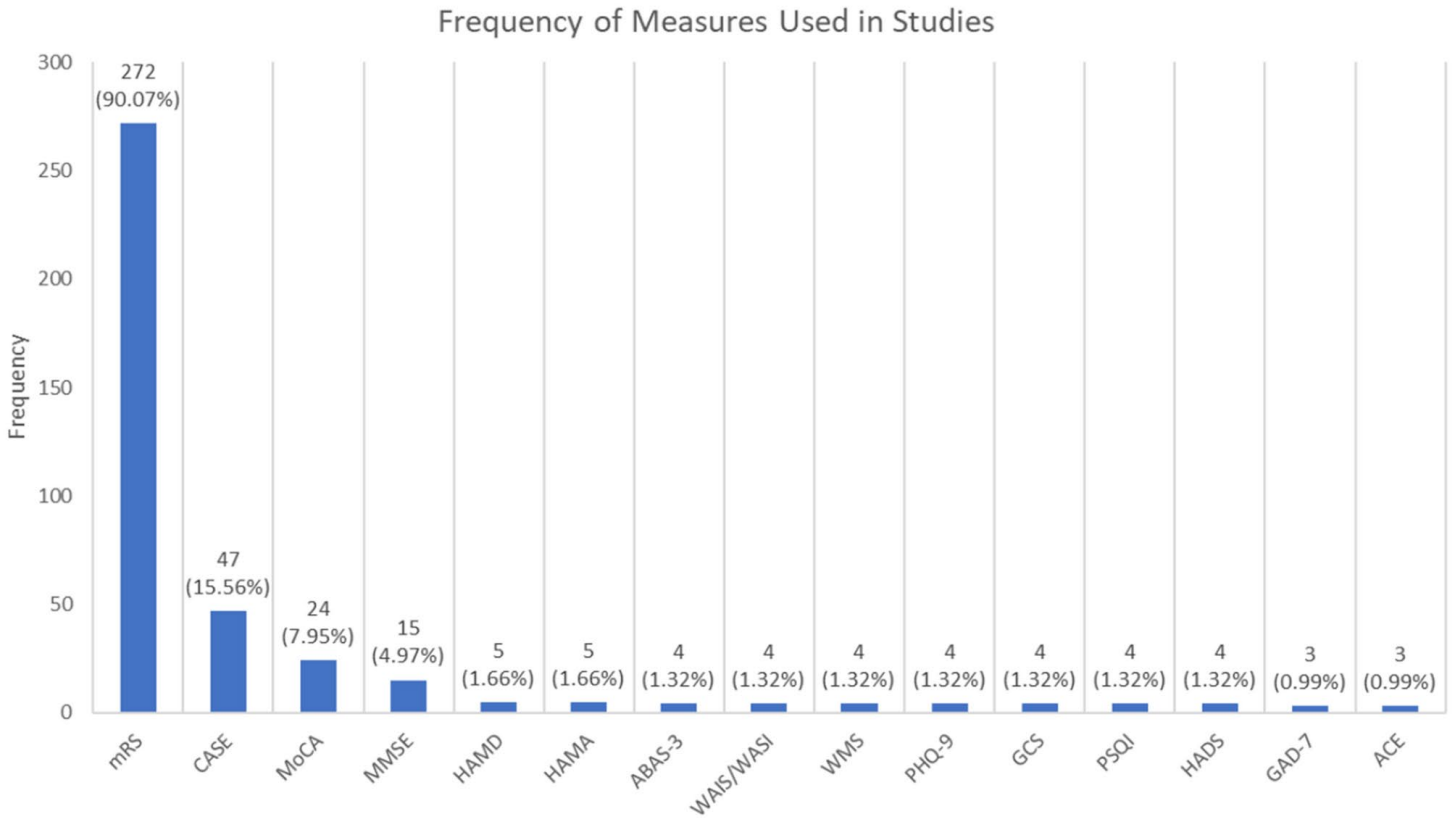
Types of quality of life/clinical measures and their applications

Figure 3 visualises the number of different measures used in AE studies in decreasing order of frequency. Measures used included mRS (Modified Rankin Scale), CASE (Clinical Assessment Scale in Autoimmune Encephalitis), MoCA (Montreal Cognitive Assessment), MMSE (Mini Mental State Examination), HAMD (Hamilton Depression Rating Scale), HAMA (Hamilton Anxiety Rating Scale). ABAS-3 (Adaptive Behaviour Assessment System), WAIS/WASI (Weschler Adult Intelligence Scale/Waschler Abbreviated Scale of Intelligence), WMS (Weschler Memory Scale), PHQ-9 (Patient Health Questionnaire), GCS (Glasgow Coma Scale), PSQI (Pittsburgh Sleep Quality Index), HADS (Hospital Anxiety and Depression Scale), GAD-7 (Generalised Anxiety Disorder) and ACE (Addenbrooke’s Cognitive Examination. Other measures were used as well, but at a lower frequency than 0.99% of the studies, refer to supplementary material 1 for the complete list.

### The mRS

The Modified Rankin Scale was used the most in the literature, at 90.07%, and as a clinical measure for disease severity. Measurements were done at numerous timepoints in the studies, including nadir, baseline upon admission, at periodic intervals defined by the study, at discharge or final follow up. Across most publications, an mRS of 2 or less was defined as a good outcome, while 2 to 6 was poor with others defining a good outcome as 3 and under. One study categorised complete recovery as mRS 0 to 1, partial recovery as 2 to 3, and disabled as 4 to 5 [29] whilst a paediatric study determined a score of 0 to be a good outcome, and 1 or more to be poor [30]. The majority of studies defined an mRS change of two or more to be a good outcome, or clinically relevant when determining intervention efficacy, with a minority defining it as a change of 1 or more as a good outcome. Other studies utilised mRS as a way to categorise patients based on severity, and whether treatment outcomes differed between severity subgroups.

### The increasing usage of CASE

CASE had the next highest frequency of utilisation at 15.56%. As a novel clinical measure specifically designed for AE, CASE appeared both in validation studies (adult, paediatric, and mixed) and as a clinical measure. In the validation studies, CASE scores were compared with mRS, functional status score (FSS), and the anti-NMDAR encephalitis one-year functional status score (NEOS). All studies found correlation between CASE scores and mRS, FSS, and NEOS scores, however Zhang et al. (2021) found no statistically significant association between CASE and relapses [31]. Three studies evaluated reliability using Cronbach’s alpha, with scores of 0.825 (item and inter-evaluator reliability), 0.83 (internal consistency), and 0.847 (internal consistency). Three studies calculated the intra-class correlation coefficient, with one finding a score of 0.96/0.98 at disease onset (inter and intra-observer reliability respectively), another finding 0.95/0.94 (inter and intra-observer reliability of total scores), and finally 0.98 (inter-evaluator reliability). Additionally, CASE was compared against MFIS, PSQI, and BDI-Fast screen in one study [32]. CASE was utilised at nadir, baseline upon admission or contact with study participants, at periodic intervals defined by the study, and discharge or final follow up. *The standard method of using the CASE score as a prognostic factor was assessing 0–4 an excellent outcome*,* 5–9 as moderate*,* and 10–27 as poor* [31, 33],. *However*,* one study defined a favourable outcome as a CASE score ≤ 2 whilst two studies used CASE to define a favourable outcome as a decrease of ≥ 5 points* [34, 35].

### Cognitive assessments

MoCA was the most frequently utilised measure of cognitive function, at 7.95% and was used as a measure for cognitive function in most studies, with the exception of one utilising it for language or speech specifically [36]. MoCA was used at nadir, baseline upon admission or contact with study participants, at periodic intervals defined by the study defined, and discharge or final follow up. Cognitive impairment was defined as a MoCA score of ≤ 25 in two studies [16, 37], with another defining mild impairment as 18–25, moderate as 10–17, and severe as < 10 [38].

MMSE appeared in 5.29% of studies as a measure of cognitive impairment, *occasionally in conjunction with a MoCA assessment.* Measurements were done at nadir, baseline upon admission or contact with study participants, at periodic intervals defined by the study, and discharge or final follow up. *The designated outcomes of MMSE varied considerably*,* with cognitive impairment being defined as MMSE < 26* [39], *MMSE < 24* [38] *and a t-MMSE of ≤ 21* [40]. In contrast, Du et al. 2022 used MMSE to define a favourable outcome as ≥ 27, or an improvement of ≥ 10 points [34]. Szots et al. (2017) designed a mental recovery score that was the difference between the lowest MMSE score, and the score at 23.4 ± 7.6 months [41].

### Lesser used measures

HAMD and HAMA (*n* = 5, 1.66%) were used as a measure for depression and anxiety in the AE cohort respectively. One study defined abnormal as HAMD > 7 while another defined mild depression as HAMD 10 to 17, and normal as 0 to 7 [36, 42]. Of the measures that appeared four times (1.32%), PHQ-9 was utilized for depression, with one study defining severity as normal (0–4), mild (5–9), moderate (10–14), moderately severe (15–19), severe ≥ 20 [38]. ABAS-3 (assessment of adaptive behaviour) had one study defining below average as ≤ 89, and another utilised the GAC and domain scores to standardise to an average of 100 (SD 15), and classified a score ≥ 120 as high, 110–119 as above average, 90–109 as average, 80–89 as below average, 71–79 as low, and ≤ 70 as extremely low [43]. WAIS/WASI and WMS (cognition) was utilised to measure processing speed and working memory, PSQI was used to measure sleep quality, GCS measured consciousness, and HADS measured anxiety and depression, with one study defining mood disturbance as a HADS score ≥ 11. These measures were used similarly to the others, at nadir, baseline upon admission or contact with study participants, at periodic intervals defined by the study, and discharge or final follow up.

The use of the health-related quality of life (HRQoL) measures were sparse, with highly validated measures such as the European Quality of Life 5 Dimensions 5 Level version (EQ-5D-5 L) appearing twice (0.66%). Neuro-QoL was also used (*n* = 2, 0.66%), followed by PedsQL, Quality of life after brain injury– overall scale (QOLIBRI-OS) and the 36-item short form survey (SF36) appearing once each (*n* = 1, 0.33%). There were 41 measures that were used in only one study, of which can be found in supplementary material 1.

Of the 272 studies utilising mRS, 267 had extractable mRS scores for AE patients. These scores mainly presented mRS as a mean or median, with some reporting IQR and range alone. External to descriptive analysis with scores, studies also reported frequency of mRS scores (e.g., eight patients with mRS of 1 at last follow up), and only whether mRS improved or deteriorated (i.e., no discrete scores). From 47 studies utilising CASE, 39 had extractable scores. The presentation of scores were similar to mRS, with the majority presenting as mean or median, and none as range or IQR alone. 18 of the 24 studies utilising MoCA and 9 of 15 for MMSE had extractable scores described as mean or median. Across all measures, there was variability in timing definitions, with a large range for follow up times and time since diagnosis (for baseline and admission time categories). Further, disclosure of comorbidities that may impact QoL or clinical measures were inconsistent. Extractable scores were available for 295 studies, and scores for each measure can be found in supplementary material 2. The original wording from the respective studies have been preserved verbatim or near verbatim to demonstrate the extent of heterogeneity.

## Discussion

The primary clinical measure used was the seven level mRS, which is capable of capturing the full functional outcome range for stroke patients and has demonstrated construct validity by showing strong correlation with stroke pathology, and convergent validity through comparative agreement with other stroke scales [44].The primary limitation is the reproducibility issue due to subjective determination between categories and the reliance on clinician judgement [45]. Regarding its application to AE, the emphasis on functional outcome does not capture the psychiatric and cognitive symptoms that are common across the spectrum of autoimmune encephalitis [46]. Cognitive impairment is a frequently cited long-term deficit in AE, with particular emphasis on memory, perceptual reasoning, attention and language [47–49]. However, Flet-Berliac et al. (2023) demonstrated that significant cognitive impairment was present in 45% of patients 2 years after diagnosis even though long-term prognosis was determined to be good as evaluated by mRS [50]. Utilisation of MMSE and MoCA can quantify the cognitive impairment as a sequelae of AE and are therefore possible adjuncts for cognitive assessment in long-term follow-up [49, 50]. Additionally, WAIS/WASI, WMS and ACE have been infrequently used as measures of cognition, but their use has remained unvalidated in AE patients [41, 48, 49]. Psychiatric symptoms such as depression and anxiety are also frequent contributors to long-term morbidity [42, 51], which are not reflected in the mRS scoring system [38].

In order to address these concerns, Lim et al. (2019) specifically designed CASE as a clinical severity tool based on the diagnostic criteria from Graus et al. (2016) (3) and seeks to be applicable to diverse AE syndromes and be more representative of the wide spectrum of AE symptoms [52]. Although several studies have shown moderate to high correlation between mRS and CASE scores [52–55], CASE compensates for the limitations of the mRS in evaluating non-motor symptoms. It is superior in its evaluation of cognitive function, particularly in the domains of language and memory [31, 56], and in its prediction of poor psychiatric outcomes [38, 53]. Cai et al. has also noted that CASE is greater at detecting changes in severity at different stages of AE, by observing changes between scores at admission and discharge [55]. Irrespective of measure validity however, Zhang et al. (2021) found the applicability mRS or CASE to be difficult in clinical practice, as the majority of patients had psychosis and their level of consciousness was difficult to evaluate [31]. Furthermore, they found that when the patient was drowsy or in stupor, memory or language problems were difficult to assess. Finally, they suggested in some cases clinicians preferred the mRS over CASE as a simpler assessment of clinical severity for AE, a sentiment that also appeared in Cai et al., (2021) [55].

The utilisation of CASE for determining long-term outcomes of AE also has its individual limitations. Notably, CASE does not sufficiently capture fatigue and sleep dysfunction in AE patients, and many patients with low CASE scores report high levels of fatigue [32, 53, 54]. Sleep disturbances are often frequent and severe, particularly in anti-NMDA, anti-LGI1 and anti-IgLON5 encephalitis [57, 58], and the associated fatigue is an accurate predictor of poor long-term QoL [51]. The CASE score also lacks precision in estimating psychosocial function, which is often significantly reduced in AE, and is an underrecognised long-term sequelae. Yokota et al. (2023) found that less than 70% of all patients had returned to their previous work or school-life five years after onset of AE, with significant declines in social QoL, including leisure, social life and sexual life [59]. Symptoms such as anxiety may impair patient function and QoL but not require medical intervention and subsequently not be noted as part of the CASE psychiatric score. Finally, Macher et al. (2023) noted CASE’s weakness in grading brainstem and cerebellar symptoms severity and progression [60], and that the greatest discrepancies between CASE and mRS occurred for patients with stiff person spectrum disorder, primarily due to the limited distribution of points (6 of 27 maximum) for CASE towards mobility scores, whilst mRS is predominantly motor/movement related.

To rectify the issues in CASE scores in capturing these QoL outcomes, additional clinical measures can be utilised at long-term follow-up to further quantify fatigue, psychosocial and cerebellar function. The PSQI has been successfully used to measure the impact of fatigue in AE [32, 57, 61], and is an important supplemental test for overall QoL at long-term follow-up. The HAMD, HAMA, HADS, PHQ-9 and GAD-7 have all been performed for the assessment of anxiety and depression in AE cohorts [55, 62, 63], which constitute a significant proportion of psychosocial burden in AE. However, none of these measures have been shown to be superior compared to another in the AE cohort. Measures of Health-Related QoL (HR-QoL) at long-term follow-up were lacking but are necessary to improve the description of disease burden holistically. Whilst SF-36 was utilised in one study [64], a major limitation was its inability to provide the basis for calculating a single measure of HR-QoL [65]. Thus, more highly validated measures such as NeuroQoL should be used, of which have demonstrated moderate to strong correlation across physical, mental and social QoL domains applicable in many neurologic conditions [66]. Regarding cerebellar function, Spatola et al. (2020) incorporated the Scale for the Assessment and Rating of Ataxia (SARA) as a measure of severity in patients with anti-mGluR1 encephalitis [67], but this scale has yet to be validated in the AE cohort.

Regarding the application of CASE scores in different cohorts, Zhou et al. (2022) and Panda et al., (2023) noted application challenges in children due to deviations in clinical presentations between adults and paediatric cohorts [68, 69]. Specifically, measuring memory deficits in younger children was more difficult [69], and some features of AE common in adults, such as seizures, are relatively rare in children [33, 68]. Thus, numerous studies have implemented and validated NEOS in the prediction of 1-year functional status for both adult and paediatric patients, most commonly for anti-NMDA encephalitis [70–72], with Nikolaus et al. (2023) suggesting that NEOS could also predict cognitive function, which is vital in the long-term outcomes of paediatric patients [70]. Measures of long-term outcomes of paediatric patients in other AEs remain scarce, necessitating further research.

Another under-recognised cohort in disease burden literature is the impact of AE on the quality of life of carers, *who can often provide important insight into a patient’s condition*,* especially when cognition or memory are impaired.* Binks et al. (2024) highlighted that approximately 50% of carers and spouses described psychological distress and recommended increased emotional support and improved neurorehabilitation for this cohort [73]. *Elevated stress levels*,* a lack of social support and uncertainty regarding the outcome of the disease could all contribute to this psychological distress. Higher levels of caregiver burden have been described when transition of care was inadequate and intended management plans were not communicated to the caregiver*,* emphasising the importance of follow-up appointments in neurology clinics* [74]. *Quantitative assessments of caregiver burden*,* such as via the Zarit Burden Interview (ZBI) would also be beneficial in assessing carer stress and identifying the need for additional social support.*

Our findings show there is no consensus for a clinical measure of a disease-free state for AE. Thus, in the absence of standardisation, studies and clinical trials have been seen to present the condition on a gradient of severity based on the impacts to quality of life, primarily utilising clinical measures for functionality (e.g., Expanded Disability Status Scale [EDSS] for physical, or neuropsychological assessments that can be used to evaluate cognitive functions such as the Modified Rankin Scale [mRS]), or utilities based HRQoL, such as the EuroQol 5 dimensions 5 levels (EQ-5D-5 L) or the Short Form 36 Health Survey (SF-36), with others measuring clinical endpoints such as seizure cessation.

Beyond the measures themselves, this study found that their application within AE literature was heterogeneous. For instance, many studies utilised these measures as a clinical endpoint to determine disease course or treatment efficacy, and variability was found in the definitions of outcomes, with ‘favourable/good’ and ‘unfavourable/poor’ outcomes not consistently comparable between studies as a result. This extended to inconsistent categorisations of disease severity by measures, predominantly the mRS, further contributing to the inability for inter-study comparison of subgroups defined by these definitions between studies. This heterogeneity extended to the extracted scores, with admission, discharge, and follow up all representing a wide range of time points within an individual’s disease course, obfuscating comparisons between studies, and reducing the viability of conducting meta-analyses on this data. For instance, a patient may be admitted upon acute onset, or chronic relapse, and different discharge criteria between global hospitals can result in measures observing different points in the disease course. Regarding nadir, presence of comorbidities separate from AE that impact the measured outcomes can confound inter-study comparisons, suggesting further studies should aim to present comorbidities comprehensively. Further, to aid future meta-analysis, a potential alternative is through the standardisation of utilising symptom onset, as this may allow researchers to categorise distinct phases of AE and investigate their associated QoL or clinical outcomes.

The quality of studies included in this systematic review had significant variability, ranging from abstracts and case studies to retrospective cohort studies, with no randomised control trials. While this limitation would detract from conclusions made on the disease burden of AE from this body of literature, it does not impact the aim of this systematic review of whether current outcome measures in clinical studies were sufficient for reporting the complete disease burden of AE. A notable limitation for the included literature was incomplete data reporting. This included stating a particular measure was utilised, but not reporting the result or specifics about its usage. As a result, it is possible that this study does not fully capture all variations of usage. There is separately the potential for selection bias, with specific measures being included in the search terms potentially disproportionately skewing measure frequency. While the search terms were a combination of commonly used clinical and quality of life measures and a comprehensive initial literature search which included cited reference searching and keyword searching, it is possible not all measures were captured. Notably, measures such as RBANS which were included in the search terms had a frequency of 1 (0.33%), while ABAS-3 that was incidentally found during full text screening, had a frequency of 4 (1.32%), suggesting high frequency measures are captured irrespective of search term inclusion.

## Conclusions

The current clinical measures do not holistically or efficiently capture the true extent of the burden of disease AE patients experience. Further, compounded by the scarcity of AE specific HRQoL measures, it presents a challenging environment where clinicians are unable to adequately assess the impact of interventions, and patients are unable to express the devastating impact AE has on their lives. A potential recommendation is the use of multiple measures to capture disease burden, with a combination of CASE for disease severity, MMSE, MoCA or ACE for cognition, HADS/HAMA for anxiety and depression, and PSIQ for sleep. The nature of QoL and clinical measures in literature are also significantly heterogeneous, impacting generalisability and comparisons between cohorts. Additionally, research involving the development of more precise measures, or the validity of combining current measures is urgently needed to assist with standardising clinical endpoints of medication trials, and burden of disease comparisons between study groups.

## Electronic supplementary material

Below is the link to the electronic supplementary material.

Supplementary Material 1

Supplementary Material 2

## Data Availability

All data collected and analysed by this systematic review are available in the supplementary material of this article.

## Abbreviations

AE: Autoimmune Encephalitis
AMPA: Alpha-amino-3-hydroxy-5-methyl-4-isoxazolepropionic acid
CASE: Clinical Assessment Scale in Autoimmune Encephalitis
CASPR: Contactin-associated protein-2
DPPX: Dipeptidyl-peptidase-like protein 6
GABA: Gamma aminobutyric acid
GAD65: Glutamic acid decarboxylase 65
IgLON5: Immunoglobulin-like cell adhesion molecule 5
LGI: 1-Leucine-rich glioma-inactivated 1
mGluR: Metabotropic glutamate receptor
MMSE: Mini Mental State Examination
MoCA: Montreal Cognitive Assessment
MOGAE: Myelin oligodendrocyte glycoprotein autoimmune encephalitis
mRS: Modified Rankin Scale
NMDA: N-methyl-D-aspartate
VGKC: Voltage-gated potassium channel
